# Understanding ART Adherence among Adolescent Girls and Young Women in Western Kenya: A Cross-Sectional Study of Barriers and Facilitators

**DOI:** 10.3390/ijerph20206922

**Published:** 2023-10-14

**Authors:** Jonathan Altamirano, Isdorah A. Odero, Mevis Omollo, Eucabeth Awuonda, Ken Ondeng’e, Jennifer L. Kang, Rasika Behl, Richard Ndivo, Michael Baiocchi, Hellen C. Barsosio, Clea C. Sarnquist

**Affiliations:** 1 Department of Epidemiology and Population Health, Stanford University School of Medicine, Stanford, CA 94305, USA; baiocchi@stanford.edu; 2 Kenya Medical Research Institute, Center for Global Health Research (KEMRI-CGHR), Kisumu 40100, Kenya; isdorah.akoth@lstmed.ac.uk (I.A.O.); corriemev@gmail.com (M.O.); eucabethawuonda@gmail.com (E.A.); kondenge@gmail.com (K.O.); rndivo@gmail.com (R.N.); hellen.barsosio@lstmed.ac.uk (H.C.B.); 3 Department of Pediatrics, Stanford University School of Medicine, Stanford, CA 94305, USA; jlkang@stanford.edu (J.L.K.); rasikab@stanford.edu (R.B.); cleas@stanford.edu (C.C.S.)

**Keywords:** HIV, intimate partner violence, treatment adherence, adolescent health, women’s health, Kenya

## Abstract

Background: HIV remains a leading cause of death for adolescent girls and young women (AGYW) in sub-Saharan Africa. This population has a high incidence of HIV and other comorbidities, such as experiencing violence, and low antiretroviral therapy (ART) adherence. To reach global HIV goals, data are needed on the specific adherence barriers for AGYW living with HIV, so interventions can be targeted effectively. Methods: Cross-sectional data were collected at urban and rural health facilities in and around Kisumu County, western Kenya, from January to June 2022, from AGYW 15–24 years of age who were living with HIV. Surveys included questions on intimate partner violence, mental health issues, food security, and orphanhood. Adherence was categorized using viral load testing where available and the Center for Adherence Support Evaluation (CASE) adherence index otherwise. Logistic regression was used to assess associations between potential explanatory variables and adherence. Findings: In total, 309 AGYW participated. AGYW with experiences of emotional violence (Odds Ratio [OR] = 1.94, 95% Confidence Interval [CI] = 1.03–3.66), moderate or severe depression (OR = 3.19, 95% CI = 1.47–6.94), and/or substance use (OR = 2.71, 95% CI = 1.24–5.92) had significantly higher odds of poor adherence when compared to AGYW without these respective experiences. Physical and sexual violence, food insecurity, and orphanhood were not associated with poor adherence in this cohort. Interpretation: Elucidating the risk factors associated with poor adherence among AGYW living with HIV allows us to identify potential targets for future interventions to improve ART adherence and HIV care outcomes. Mental health and violence prevention interventions, including combination interventions, may prove to be promising approaches.

## 1. Introduction

HIV is a major cause of death and disability for adolescent girls and young women (AGYW) aged 15–24 in many parts of sub-Saharan Africa [1]. In Kenya, and globally, this age group has both a high HIV incidence and low rates of antiretroviral therapy (ART) adherence, despite ART becoming more widely available [2]. This population is failing to meet the Joint United Nations Programme on HIV/AIDS (UNAIDS) 95-95-95 goals; that is, that 95% of people living with HIV will know their HIV status, that 95% of people who know their status will be on ART treatment, and that 95% of people on treatment will have a suppressed viral load. Furthermore, failure in this and a few other sub-populations may put the global 95-95-95 goals at risk [3].

Risk factors for poor ART adherence among AGYW living with HIV include being a woman, experiencing substance abuse, poor mental health, and/or poverty, being an orphan, and experiencing or witnessing violence. Challenges to adherence are often intersectional and specific to regions, communities, and individuals. For example, a 2015 review article on the risk factors for poor adolescent adherence in low- and middle-income countries found only 15 rigorous studies, but 35 risk factors within those 15 [4].

There is also a dearth of evidence-based interventions for ART adherence aimed specifically at AGYW. A 2018 review paper of interventions in low- and middle-income countries found only eight that had adolescents as one of the target populations; only two focused on adolescents and several of the included studies had significant methodological issues [5]. This represents a research gap not only in global HIV care and treatment, but also missed opportunities in HIV prevention, as low viral loads lead to decreased transmission.

Studies have shown a complex relationship between ART adherence and gender. Women sometimes, but not always, have lower adherence than men. These differences are likely driven by different adherence barriers and facilitators in different communities [6]. Furthermore, while gender-specific ART adherence interventions seem to be effective, based on a meta-analysis of 15 studies, there is a notable lack of evidence on gender-specific interventions targeting adolescents and young women [7]. To target such interventions appropriately, baseline data on the drivers of non-adherence in this population are needed.

The COVID-19 pandemic has further limited our understanding of the current ART adherence needs and challenges. The pandemic-related stress and mobility restrictions, mostly implemented in 2020–2021, exacerbated the known risk factors for poor adherence, including poor mental health, poverty, violence, and substance abuse [8,9,10]. Therefore, there is an urgent need to understand and address the current obstacles to ART adherence in AGYW living with HIV, especially those who are most vulnerable.

In the context of the variety of risk factors for ART non-adherence and the unknown effects of the COVID-19 pandemic and related restrictions on ART adherence, this study aimed to assess the barriers to ART adherence among AGYW living with HIV in western Kenya in 2022. The results of this study should help to effectively target future interventions in this population.

## 2. Methods

### 2.1. Study Site and Recruitment

This cross-sectional study was conducted at urban and rural health facilities in and around Kisumu County in western Kenya from January to June 2022. Eligible participants included AGYW aged 15–24 with a laboratory-confirmed HIV diagnosis at least six months prior to the beginning of the study. Potential participants were identified at health facilities and invited to participate by the study staff. Recruitment efforts were supported by adolescent health clinic peer leaders and clinic nursing staff, who helped the study staff approach eligible participants. AGYW were excluded from the study if they had significant mental or physical deficits that impaired their ability to consent to participation, such as severe learning impairment or physical or mental illness. The study staff scheduled appointments with potential participants to obtain written informed consent for adults or verbal assent and opt-out parent/guardian written informed consent for minors. This study was approved by the Stanford University School of Medicine’s Institutional Review Board (IRB #51391), the Kenya Medical Research Institute’s Scientific and Ethics Review Unit (SERU/CGHR/192/4130), and the County Government of Kisumu, Department of Health, Jaramogi Oginga Odinga Teaching and Referral Hospital Institutional Scientific Ethics Review Committee (IERC/JOOTRH/535/21).

### 2.2. Data Collection

After consent and enrollment, electronic surveys were administered by staff using tablets. The data were entered and managed using REDCap (Research Electronic Data Capture version 13.9.1). The Stanford REDCap platform (http://redcap.stanford.edu (accessed on 29 September 2023)) was developed and is operated by the Stanford Medicine Research Technology team. The REDCap platform services at Stanford are subsidized by (a) Stanford School of Medicine Research Office and (b) the National Center for Research Resources and the National Center for Advancing Translational Sciences, National Institutes of Health, through grant UL1 TR001085. The survey elements were pulled from pre-existing validated instruments and included: (1) the Demographic and Health Survey Domestic Violence Module, to ask questions regarding intimate partner violence (IPV), including emotional, physical, and sexual violence, as well as to ask about non-partner violence [11], (2) the nine-item Patient Health Questionnaire (PHQ-9) [12], to assess the prevalence of major depressive disorder and the seven-item Generalized Anxiety Disorder (GAD-7) [13] scale to measure anxiety, (3) the Child and Youth Resilience Measure (CYRM-R) [14] to assess resilience, and (4) the Center for Adherence Support and Evaluation (CASE) self-reported index, a three-item survey instrument used to assess ART adherence [15]. The CASE index and PHQ-9 have both been validated in youth in Kenya, and the GAD-9 has been validated in adults in Kenya. Prior research among Kenyan adolescents in Nairobi has identified the CASE index as a reasonable substitute for viral load testing [16], a result which mirrors the use of the CASE index in other settings [17]. Medical chart data were also abstracted to collect viral load data from the most recent prior routine HIV clinic visit.

### 2.3. Determination of ART Adherence

The original protocol for this study relied on HIV viral load testing as the key outcome measure. At the time that protocol was written, such testing was widely available in this region. However, due to the COVID-19 pandemic disrupting healthcare access and demand, limited availability of reagents and supply chain challenges, as well as changes in viral load reimbursement and practices in Kenya, recent viral load data were not available for many participants. Thus, we modified our main outcome measure. Recent literature suggests a need to combine multiple metrics, specifically for adolescents in sub-Saharan Africa, as the data quality for adolescents may be especially complicated [18]. Based on this literature, we decided to categorize the participants as having poor adherence if they received a poor score with the CASE index or, when available, if their most recent viral load within six months of the study visit was above a certain threshold. In the case of disagreement between the two methods, the participants were categorized as having poor ART adherence. Table 1 shows the decision making used to categorize participants as having good or poor adherence.

Two thresholds were used for the viral load data: ≥200 copies/mL as per the U.S. Centers for Disease Control and Prevention (CDC) guidelines and ≥1000 copies/mL as per the World Health Organization (WHO) guidelines to reflect both U.S. and global standards. Separate analyses were run using each viral load threshold in combination with the CASE index. The results for the model using the CDC’s viral load threshold can be found below in the main text, while the results of the model using the WHO’s guidelines can be found in the Appendix A.

### 2.4. Data Analysis

The data were analyzed using unconditional logistic regression. Odds ratios (OR) with 95% confidence intervals (CI) were used to assess the association between poor ART adherence and five measures of interest, including types of violence, specifically (a) physical IPV, (b) emotional IPV, and (c) sexual violence, which was a combination of partner sexual violence and non-partner sexual violence, as well as (d) depression and (e) substance use. A separate model was run for each measure of interest after evaluating and adjusting for potential confounding factors based on previous literature [4]. Confounders included clinic type (urban vs. rural), age in years, household size, maternal education (primary education and below vs. secondary education and above), orphanhood status (both parents alive vs. only one parent vs. no parents alive), food insecurity in the last two weeks, and transactional sex (defined as sex in exchange for money, food, gifts, or favors).

The model for sexual violence was adjusted for urban vs. rural clinic, reported transactional sex, and age in years. The models for emotional and physical violence, substance use, and depression status were adjusted for urban vs. rural clinic, reported ever having had sex, and age in years. Household size, maternal education, orphanhood status, and food insecurity were not associated with violence or ART adherence status and were excluded from the final models as a result. Each final model was run using both the CDC and WHO viral load thresholds, 200 copies/mL and 1000 copies/mL, respectively. All the statistical analyses were performed using SAS software (version 9.4, SAS Institute Inc., Cary, NC, USA).

### 2.5. Sensitivity Analysis

In order to understand how much our results would differ if we were more stringent with our definition of poor ART adherence, we additionally ran a model with an adherence measure that prioritized viral load measures. First, the participants were categorized as having poor adherence if their most recent viral load measure was above the CDC threshold (≥200 copies/mL). From there, all the participants without a recorded viral load within six months of their study visit were categorized as having good or poor adherence using their responses to the CASE index.

## 3. Results

### 3.1. Participant Demographics

Table 2 displays the demographic characteristics of the 309 AGYW who were recruited for the study. The majority of participants were recruited from rural clinics (*n* = 185, 59.87%) rather than urban clinics, with a median age of 21.0 (Interquartile Range [IQR]: 18.0–23.0) and a median household size of 6.0 people (IQR: 4.0–7.0). The majority of the participants reported their mother’s highest level of education as being primary school or below (*n* = 132, 42.72%), although a quarter of participants (*n* = 85, 27.51%) were unaware of their mother’s highest level of education. Only a third of the study participants still had both parents alive (*n* = 106, 34.3%); a quarter reported that both parents were deceased (*n* = 75, 24.27%). Almost half of the study participants reported skipping at least one meal in the last two weeks as a result of food insecurity (*n* = 128, 41.43%). Most participants reported having had a boyfriend in the last year (*n* = 232, 76.32%). Finally, only 44 (14.33%) participants reported the use of either drugs or alcohol.

### 3.2. Sexual History and Rates of Violence

Just over 70% of the participants (*n* = 216, Table 3) reported ever having had sex, with 60 of these participants (27.8%) additionally reporting that they had exchanged sex for money, food, gifts, or other favors. Rates of violence were high in this population, with just over a quarter of these 15–24-year olds reporting physical IPV (*n* = 82, 26.89%), nearly 35% reporting sexual violence in the last year (*n* = 108, 34.95%), and almost 40% reporting emotional IPV (*n* = 120, 38.83%). Of those reporting sexual violence, non-partner sexual violence was more commonly reported than sexual IPV, at 27% (*n* = 84) and 18% (*n* = 56), respectively.

The risk of each type of violence increased with age. Among our 60 participants under 18, 13.3% reported physical IPV (*n* = 8), 16.7% reported emotional IPV (*n* = 10), and 30% reported sexual violence (*n* = 18). In comparison, among the 122 participants aged 18–22 years old, 18.9% reported physical IPV (*n* = 23), 32.0% reported emotional IPV, and 31.2% reported sexual violence, while, among the 127 participants aged 23–24 years old, 40.2% reported physical IPV (*n* = 51), 55.9% reported emotional IPV (*n* = 71), and 40.9% reported sexual violence (*n* = 52).

### 3.3. Mental Health Measures

In total, 14% (*n* = 42) of the participants had moderate to severe depression, while an additional third scored in the mild depression range (*n* = 97, 33.11%), as shown in Table 4. Rates of anxiety were lower than rates of depression, with only 6% of the participants scoring in the moderate to high anxiety range (*n* = 17), and almost 30% scoring in the mild anxiety range (*n* = 86). Resilience scores were high, with a median score of 47.0 (IQR: 43–49) out of a possible score of 51.

### 3.4. Adherence Measures

Only 48 of the 309 AGYW (15.5%) had a viral load reading within six months of their enrollment in the study (Table 1). Of these, 8.3% (*n* = 4) had poor adherence as defined by the CDC and 6.3% (*n* = 3) had poor adherence as defined by the WHO. The CASE Adherence Index was successfully completed for all but one AGYW in the cohort and identified 21.75% (*n* = 67) participants with poor adherence. Three participants were identified as having poor adherence using the WHO cutoff (≥1000 copies/mL) that were not identified by the CASE index, and one additional participant was identified as having poor adherence using the CDC cutoff (≥200 copies/mL) that was missed by the CASE index. As a result, the final model using the WHO cutoff included 70 participants with poor adherence (22.7%), while the model using the CDC cutoff included 71 participants with poor adherence (23.0%). For more details on the breakdown of the CASE index results stratified by the viral load measures, please see Table A1.

### 3.5. ART Adherence Models: CDC Viral Load Cut-Off plus CASE Index Model

Table 5 shows the association between each type of violence (sexual, emotional, or physical), substance use, and depression with poor adherence as defined by the CASE index and the CDC. AGYW with moderate or severe depression had more than three times the odds of poor adherence when compared to those with no or minimal depression (OR = 3.19, 95% CI = 1.47–6.94). Those reporting any experiences of emotional IPV also had almost twice the odds of poor adherence when compared to those without reported emotional IPV (OR = 1.94, 95% CI = 1.03–3.66). The participants with reported substance use also had more than twice the odds of poor ART adherence when compared to those without reported substance use (OR = 2.71, 95% CI = 1.24–5.92). There were no statistically significant differences in the odds of poor adherence between participants with mild depression compared to those without (OR = 1.44, 95% CI = 0.74–2.79), between those with any reported experiences of physical violence compared to those without physical violence (OR = 1.67, 95% CI = 0.88–3.15), and between those who reported any sexual violence in the last year compared to those without reported sexual violence (OR = 1.14, 95% CI = 0.62–2.10).

### 3.6. Sensitivity Analysis for Adherence Measures (Table A1)

The results of the sensitivity analysis prioritizing the viral load in the determination of poor ART adherence can be found in the Appendix A (Table A2). In brief, this model found comparable results to the more inclusive model presented in Table 5. The participants with experiences of emotional violence, with reported substance use, and/or with moderate-to-severe depression all showed significantly increased odds of poor adherence when compared to those without these experiences.

## 4. Discussion

In this study of AGYW living with HIV in western Kenya, we found significant correlations between experiencing emotional IPV, having moderate or severe depression, and/or substance use and poorer adherence to ART regimens. We did not, however, find correlations between orphanhood, food insecurity, and physical or sexual violence and adherence, in contrast to findings from research on other populations.

Multiple studies have found a link between poor mental health, especially depression, and poor adherence in adolescents globally and in sub-Saharan Africa [19,20,21,22,23]. This link has been documented in Kenya as well, where, for example, being depressed and adhering to medication were significantly correlated in a cohort of mixed-gender adolescents [20]. Despite this well-recognized connection, there is a paucity of rigorously evaluated mental health interventions for AGYW living with HIV/AIDS, and multiple calls to action for more work have been issued in this area to improve global HIV outcomes [24,25].

Experiencing violence, especially stratified into different types of violence and violence beyond IPV, has rarely been included as a potential risk factor for poor adherence in studies of AGYW. When it has been included, however, it has often proven to be a significant predictor of poor adherence across several countries. In Malawi, for example, among both male and female adolescents (aged 10–19) living with HIV, exposure to violence in the home was negatively associated with ART adherence [26]. In Zambia, experiencing violence in the past year, especially frequent psychological/emotional violence, was shown to be associated with viral load failure among male and female youth aged 15–24 [27]. In South Africa, there have been mixed results about the role of violence in AGYW’s adherence. Recent studies have reported that both IPV (of any type) and sexual abuse independently negatively impact ART adherence among adolescents living with HIV [28], but other analyses have found no significant impact among AGYW [29]. In Kenya specifically, IPV has been reported as a significant driver of poor ART adherence for the general population of adult women [30].

Similar to many studies conducted among AGYW living with HIV, we identified substance use as a risk factor for increased odds of poor ART adherence [3,7,8,9]. While only 14% of our study population reported alcohol drinking or illicit drug use, it is plausible that this sub-population may have unique needs that must be addressed in order to most effectively improve their medication adherence. More research is needed to understand the unique experiences of AGYW living with HIV that also regularly engage in substance use.

In this cohort, we did not observe significant correlations in the two other types of violence we measured, physical IPV and sexual violence, nor in food insecurity or being an orphan. There are several possible explanations for this. The first is that these are not important drivers in this community and population, as adherence to ART is often complex and driven by many factors, as discussed above [4]. A social-ecological approach to targeting interventions is likely necessary. A second plausible explanation is that our sample size may have been too small to detect the importance of these factors, as several of them showed non-significant trends toward affecting adherence.

Despite not finding an association between sexual and/or physical violence and poor ART adherence, we did find that over a third of these AGYW reported prior-year sexual violence, and nearly a third reported physical violence. These results mirror recent estimates released by the 2022 Kenya Demographic and Health Survey. While the results reported in the Demographic and Health Survey were not stratified by HIV status, the survey found that 35% of women in Kisumu who reported ever having a husband or an intimate partner also reported experiencing emotional, physical, or sexual violence in the last twelve months [31]. Since experiencing violence, especially in adolescence, has a wide range of negative health and behavioral outcomes [29,32,33], our findings suggest a significant need for interventions to reduce violence in this population.

### Limitations

This study had several key limitations. First, as a result of the ongoing COVID-19 pandemic, as well as a changing viral load testing reimbursement landscape, viral load data could not be fully relied upon to assess adherence to ART. The viral load data for the 48 participants who did have a viral load sample collected within six months of their study interview did identify additional participants with poor adherence, depending on which of the two cutoff models we used to define poor adherence, suggesting that our estimate for poor ART adherence in this population was likely an underestimate. Second, the number of participants who were non-adherent to their ART medications was lower than expected based on previous research from other parts of Kenya, likely reflecting good access to services, even in more rural areas surrounding Kisumu. While we were pleased to see a relatively high adherence, this nonetheless led to smaller numbers than expected for the study, and may explain some of the unexpected findings. Third, small numbers reporting some of the potential risk factors may also have prevented us from detecting true relationships. Finally, we did not have information on whether HIV transmission to these AGYW was vertical, from parent-to-child, or horizontal, such as through sexual contact. Prior literature suggests that the majority of HIV infections among 15–24-year olds in Kenya are the result of sexual transmission [34]. However, these subpopulations may have different risks factors for poor adherence or different underlying characteristics that should be addressed in future research with a larger sample size.

## 5. Conclusions

Understanding what experiences and risk factors are significantly associated with sub-optimal HIV medication adherence allows us to identify potential targets for future interventions to improve HIV care outcomes. In this population of AGYW in western Kenya, mental health challenges and violence were found to be key drivers of low adherence and may prove to be promising avenues for intervention. These findings are consistent with the literature in this area, and it is likely that they are applicable to settings beyond western Kenya and sub-Saharan Africa. Given the global lack of evidence-based interventions to improve adherence specifically for AGYW and the range of different risk factors associated with low adherence in different populations, locally tailored and adapted combination interventions are needed.

## Figures and Tables

**Table 1:** Classification of HIV ART adherence by viral load and CASE Adherence Index results.

	CASE Adherence Index Results	
Viral Load Results	Poor Adherence	Good Adherence
Poor Adherence (≥200 copies of HIV/mL)	Poor ART Adherence	Poor ART Adherence
Good Adherence (<200 copies of HIV/mL)	Poor ART Adherence	Good ART Adherence

**Table 2:** Participant demographic information.

Variables	No. (%) *n* = 309
Clinic Type	
Urban	124 (40.13)
Rural	185 (59.87)
Age Group	
Under 18	60 (19.42)
18–22	122 (39.48)
23+	127 (41.1)
Age in years (Median, IQR)	21.0 (18.0–23.0)
Household Size (Median, IQR)	6.0 (4.0–7.0)
Maternal Education	
No Education	2 (0.65)
Primary School, Incomplete	37 (11.97)
Primary School, Complete	93 (30.1)
Secondary School, Incomplete	20 (6.47)
Secondary School, Complete	50 (16.18)
Post-secondary School, Complete	22 (7.12)
Unknown ^a^	85 (27.51)
Living Parents	
Both Parents Alive	106 (34.3)
Only Mother Alive	99 (32.04)
Only Father Alive	29 (9.39)
Neither Parent Alive	75 (24.27)
Self-reported Food Insecurity	
Yes	128 (41.42)
No	181 (58.58)
Boyfriend in last 12 months	
Yes	232 (76.32)
No	72 (23.68)
Missing Current Boyfriend ^b^	5
Self-reported substance use	
Never	263 (85.67)
Ever	44 (14.33)
Missing substance use ^b^	2
CASE Adherence Index	
Poor Adherence	67 (21.75)
Good Adherence	241 (78.25)
Missing Adherence ^a^	1
Viral Load Data ^c^	
<200 copies/mL	44 (91.7%)
≥200 copies/mL	4 (8.3%)
≥1000 copies/mL	3 (6.3%)
Missing viral load data w/in last six months ^a^	261

^a^ Participants reported not knowing their mother’s education level; ^b^ Missing values are not included in calculated proportions. ^c^ Viral load data were extracted for all participants with a clinic visit within six months of their interview date. Interruption of routine testing during the COVID-19 pandemic resulted in low testing rates.

**Table 3:** Participant sexual history and reported experience of violence.

Variables	No. (%) *n* = 309
Ever had sex	
Yes	216 (70.59)
No	90 (29.41)
Missing ever had sex ^a^	3
Ever had transactional sex ^b^	
Yes	60 (27.78)
No	156 (72.22)
Physical Intimate Partner Violence	
Reported	82 (26.89)
Missing ^a^	4
Emotional Intimate Partner Violence	
Reported	120 (38.83)
Any Sexual Violence	
Reported	108 (34.95)
Missing ^a^	1
Non-partner Sexual Violence	
Reported	84 (27.27)
Missing ^a^	1
Partner Sexual Violence	
Reported	56 (18.18)
Missing ^a^	1
Sexual Assault	
Any completed sexual assault	91 (29.55)
Partner sexual assault	44 (14.29)
Non-partner sexual assault	63 (20.45)
Missing completed sexual assault ^a^	1
Reported Perpetrators of Non-partner Sexual Assault	
Authority Figure	6 (9.52)
Family Member or Relative	12 (19.05)
Friend	10 (15.87)
Neighbor	14 (22.22)
Other	7 (11.11)
Don’t Know	12 (19.05)
Refused	2 (0.65)

^a^ Missing values are not included in calculated proportions; ^b^ Transactional sex was defined as sex in exchange for money, food, gifts, or favors. Proportions for the transactional sex variable are out of participants who reported having ever had sex (*n* = 216).

**Table 4:** Participant mental health measures.

Variables	No. (%) *n* = 309
Mental Health Measure	
PHQ-9 Depression Score	
No/Minimal Depression (0–4)	154 (52.56)
Mild Depression (5–9)	97 (33.11)
Moderate Depression (10–14)	30 (10.24)
Moderately Severe Depression (15–19)	9 (3.07)
Severe Depression (20–27)	3 (1.02)
Missing ^a^	16
GAD Anxiety Score	
No/Minimal Anxiety (0–4)	184 (64.11)
Mild Anxiety (5–9)	86 (29.97)
Moderate Anxiety (10–14)	10 (3.48)
Severe Anxiety (15–21)	7 (2.44)
Missing ^a^	22
CYRM-R Score (Median, IQR)	47 (43–49)

^a^ Missing values are not included in calculated proportions.

**Table 5:** Adjusted odds ratios (OR) and 95% confidence intervals (CI) for the association between physical, emotional, and sexual violence, depression, substance use, and risk of poor HIV medication adherence (CASE Adherence Index or Viral Load > 200 copies/mL).

	Good Adherence		Poor Adherence			
	*n*	%	N	%	Crude OR (95% CI)	Adjusted OR (95% CI)
No Sexual Violence ^†^	156	66.10	43	60.56	(Ref)	(Ref)
Any Sexual Violence ^†^	80	33.90	28	39.44	1.27 (0.73–2.19)	1.14 (0.62–2.10)
No Emotional Violence ^††^	151	63.71	37	52.11	(Ref)	(Ref)
Any Emotional Violence ^††^	86	36.29	34	47.89	1.61 (0.94–2.76)	1.94 (1.03–3.66)
No Physical Violence ^††^	178	76.07	45	64.29	(Ref)	(Ref)
Any Physical Violence ^††^	56	23.93	25	35.71	1.77 (0.99–3.13)	1.67 (0.88–3.15)
No Substance Use ^††^	211	89.79	51	71.83	(Ref)	(Ref)
Substance Use ^††^	24	10.21	20	28.17	3.45 (1.77–6.72)	2.71 (1.24–5.92)
PHQ: Minimal ^††^	127	56.44	27	40.30	(Ref)	(Ref)
PHQ: Mild ^††^	75	33.33	21	31.34	1.32 (0.70–2.49)	1.44 (0.74–2.79)
PHQ: Moderate+ ^††^	23	10.22	19	28.36	3.89 (1.86–8.11)	3.19 (1.47–6.94)

^†^ Adjusted for urban vs. rural clinic, reported participation in transactional sex, and age in years; ^††^ Adjusted for urban vs. rural clinic, reported having had sex in the past, and age in years.

## Data Availability

The data used in this study are not publicly available. Participant consent and assent forms included language preventing the sharing of these data.

## Appendix A

**Table A1:** CASE Adherence Index results stratified by HIV viral load measure.

	Viral Load > 200 copies/mL: Poor ART Adherence *n* = 4	Viral Load < 200 copies/mL: Good ART Adherence *n* = 44	Viral Load Missing: Missing ART Adherence *n* = 261
CASE Adherence Results			
Poor ART Adherence	0 (0%)	3 (6.82%)	64 (23.46%)
Good ART Adherence	4 (100%)	41 (93.18%)	199 (76.54%)
Missing CASE Index	0	0	1

**Table A2:** Adjusted odds ratios (OR) and 95% confidence intervals (CI) for the association between physical, emotional, and sexual violence, depression, and substance use on the risk of poor HIV medication adherence: sensitivity analysis of combined adherence measures.

	Original Model ^a^	Sensitivity Analysis ^b^
	Adjusted OR (95% CI)	Adjusted OR (95% CI)
No Sexual Violence ^†^	(Ref)	(Ref)
Any Sexual Violence ^†^	1.14 (0.62–2.10)	1.32 (0.72–2.44)
No Emotional Violence ^††^	(Ref)	(Ref)
Any Emotional Violence ^††^	1.94 (1.03–3.66)	2.22 (1.17–4.21)
No Physical Violence ^††^	(Ref)	(Ref)
Any Physical Violence ^††^	1.67 (0.88–3.15)	1.87 (0.999–3.53)
No Substance Use ^††^	(Ref)	(Ref)
Substance Use ^††^	2.70 (1.24–5.92)	2.30 (1.05–5.05)
PHQ: Minimal ^††^	(Ref)	(Ref)
PHQ: Mild ^††^	1.44 (0.74–2.79)	1.43 (0.74–2.77)
PHQ: Moderate+ ^††^	3.19 (1.47–6.94)	2.36 (1.08–5.20)

^a^ Original results presented in Table 4, where participants were considered as having poor adherence if they had a poor CASE index score or if their viral load ≥ 200 copies/mL within 6 months of their study visit; ^b^ Adherence measure prioritizes viral load first (≥200 copies/mL for poor adherence) and only considers CASE index when no viral load was measured within 6 months of study visit; ^†^ Adjusted for urban vs. rural clinic, reported participation in transactional sex, and age in years; ^††^ Adjusted for urban vs. rural clinic, reported having had sex in the past, and age in years.

**Table A3:** Adjusted odds ratios (OR) and 95% confidence intervals (CI) for the association between physical, emotional, and sexual violence, depression, substance use, and risk of poor HIV medication adherence (CASE Adherence Index or viral load > 1000 copies/mL).

	Good Adherence		Poor Adherence			
	*n*	%	*n*	%	Crude OR (95% CI)	Adjusted OR (95% CI)
No Sexual Violence ^†^	157	66.24	42	60.00	(Ref)	(Ref)
Any Sexual Violence ^†^	80	33.76	28	40.00	1.31	1.16 (0.63–2.14)
No Emotional Violence ^††^	151	63.45	37	52.86	(Ref)	(Ref)
Any Emotional Violence ^††^	87	36.55	33	47.14	1.50 (0.90–2.65)	1.86 (0.99–3.49)
No Physical Violence ^††^	178	75.74	45	65.22	(Ref)	(Ref)
Any Physical Violence ^††^	57	24.26	24	34.78	1.67 (0.93–2.97)	1.60 (0.84–3.02)
No Substance Use ^††^	211	89.41	51	72.86	(Ref)	(Ref)
Substance Use ^††^	25	10.59	19	27.14	3.14 (1.61–6.15)	2.40 (1.09–5.29)
PHQ: Minimal ^††^	127	56.19	27	40.91	(Ref)	(Ref)
PHQ: Mild ^††^	76	33.63	20	30.30	1.24 (0.65–2.36)	1.35 (0.69–2.63)
PHQ: Moderate+ ^††^	23	10.18	19	28.79	3.89 (1.86–8.11)	3.23 (1.24–6.00)

^†^ Adjusted for urban vs. rural clinic, reported participation in transactional sex, and age in years; ^††^ Adjusted for urban vs. rural clinic, reported having had sex in the past, and age in years.

### Appendix A.1. Table A3 Model: WHO Viral Load Cut-Off plus CASE Index

Table A3 shows the association between violence (sexual, emotional, and physical), substance use, and depression with poor adherence as defined by the CASE index and WHO. AGYW with moderate or severe depression showed over three times the odds of poor adherence when compared to those with no or minimal depression ([OR] = 3.23, 95% [CI] = 1.24–6.00). The participants with reported substance use also had more than twice the odds of poor ART adherence when compared to those without reported substance use (OR = 2.40, 95% CI = 1.09–5.29). There were no statistically significant differences in odds of poor adherence between the participants with mild depression compared to those with no or minimal depression (OR = 1.35, 95% CI = 0.69–2.63), or between the participants reporting experiences of physical, emotional, or sexual violence compared to those without experiences of violence (OR = 1.60, 95% CI = 0.84–3.02; OR = 1.86, 95% CI = 0.99–3.49; and OR = 1.16, 95% CI = 0.63–2.14, respectively).
